# The effects of the healthcare line in a stroke unit: three years' experience of a center in the Northeast of Brazil

**DOI:** 10.1055/s-0043-1770350

**Published:** 2023-08-30

**Authors:** Deborah Moreira Rangel, Anna Karuza Nogueira Feitosa, Flaviane Melo Araújo, Mara Cibelly da Silva Pinheiro, Alan Alves de Lima Cidrão

**Affiliations:** 1 Hospital Geral de Fortaleza, Fortaleza CE, Brazil.; 2 Instituto de Saúde e Gestão Hospitalar, Hospital Regional do Sertão Central, Serviço de AVC, Quixeramobim CE, Brazil.

**Keywords:** Cerebrovascular Disorders, Mortality, Risk Factors, Stroke, Thrombolytic Therapy, Transtornos Cerebrovasculares, Mortalidade, Fatores de Risco, Acidente Vascular Cerebral, Terapia Trombolítica

## Abstract

**Background** Treatment at an organized stroke unit center (SUC) improves survival after stroke. Stroke mortality has decreased worldwide in recent decades.

**Objective** This study shows the experience of a SUC in the Northeast of Brazil, comparing its first, second, and third years.

**Methods** We compared data on the SUC prospectively collected from 31 July 2018 to 31 July 2019 (year 1), August 1 ^st^ , 2019, to July 31 ^st^ , 2020 (year 2), and August 1 ^st^ to July 31 ^st^ , 2021 (year 3).

**Results** There was an expertise evolution through the years, with good outcomes in spite of the coronavirus disease 2019 pandemic in the 3 ^rd^ year. Also, in the 1 ^st^ year, the median (interquartile range) door-to-needle time was 39.5 (29.5–60.8) minutes evolving to 22 (17–30) minutes, and then to 17 (14–22) minutes in the last year.

**Conclusion** This was the first report on a SUC's outcome in the Brazil's Central Arid Northeast countryside, and it shows the improvement in care for patients with stroke through an effective healthcare line.

## Resumo

**Antecedentes** O tratamento em um centro organizado com Unidade de acidente vascular cerebral (AVC) melhora a sobrevida após o AVC. A mortalidade por AVC diminuiu em todo o mundo nas últimas décadas.

**Objetivo** Este estudo mostra a experiência de um centro de AVC no Nordeste brasileiro, comparando o primeiro, segundo e terceiro anos do serviço.

**Métodos** Nós comparamos dados coletados prospectivamente na Unidade de AVC de 31 de julho de 2018 a 31 de julho 2019 (ano 1), 1° de agosto de 2019 a 31 de julho de 2020 (ano 2) e 1° de agosto a 31 de julho de 2021 (ano 3).

**Resultados** Houve uma evolução na conhecimento especializado ao longo dos anos, com bons desfechos apesar da pandemia de *coronavirus disease 2019* no terceiro ano. Além disso, no primeiro ano a mediana do tempo porta–agulha foi de 39.5 (29.5–60.8) minutos, evoluindo para 22 (17–30) minutos, e então 17 (14–22) minutos no último ano.

**Conclusão** Este foi o primeiro relato sobre o desempenho de um serviço de AVC do interior do Nordeste brasileiro e evidencia a melhoria assistencial aos pacientes com AVC por meio de uma efetiva linha de cuidado em saúde.

## INTRODUCTION

Stroke is a major national health problem in Brazil. Recently, data has shown that cardiovascular disease continues to be the main cause of mortality, as per the Brazilian Institute of Geography and Statistics (IBGE). 1

There has been a downward trend in stroke mortality, mainly in the southern and southeastern regions. 2 3

One of the most effective interventions after stroke is patient referral to an organized Stroke Unit Care (SUC) during the acute phase, with evidence indicating that it increases independence, survival, and rates of living at home by 12 months. The SUC improves functional outcomes and decreases the length of hospital stay when compared to patients admitted elsewhere. 4 5 6

The goal of the present study is to show the experience of a SUC in the Brazilian Northeast countryside, comparing its first, second, and third years of service.

## METHODS

### Study setting and participants

Data on consecutive stroke admissions to the SUC were prospectively collected from July 31 ^st^ , 2018, to July 31 ^st^ , 2019, which was considered year one; August 1 ^st^ , 2019, to July 31 ^st^ , 2020, year two, and August 1 ^st^ , 2020, to July 31 ^st^ , 2021, year three.

The inclusion criteria used were patients treated at a SUC; diagnosis of stroke, transient ischemic attack (TIA), or cerebral venous thrombosis; and less than 72 h of symptom onset.

The World Health Organization's (WHO) definition of stroke 7 was used; however, patients diagnosed with stroke but with symptom resolution within 24h due to treatment with intravenous thrombolysis were still classified as having a stroke.

### Stroke unit

Our stroke unit is localized in the Brazil's Central Arid Northeast countryside, and it is composed by a multidisciplinary team of neurologists, nurses, nursing assistants, physiotherapists, occupational therapists, phonoaudiologists, nutritionists, clinical pharmacists. Neurosurgeons, psychologists, and social service workers are available on demand.

The unit is composed by 10 monitored hospital beds and is reference to 20 countryside cities in the area.

Year one was integrated by neurologists but also clinical physicians on duty with daily neurologist survey (nowadays, all physicians on duty are neurologists). At that time, there were no neurosurgeons in the hospital. Surgical patients were transferred to another support hospital. Thrombolysis was done in the SUC, after the patient returned from computed tomography (CT).

Nowadays, since year two, there are neurosurgeons in the stroke team, available when necessary, and thrombolyses are initiated in the tomography room.

The dose of recombinant tissue plasminogen activator (tPA) used in our protocol is 0.9 mg/kg (maximum 90 mg).

### Outcomes and measures

The primary outcome in this study was door-to-needle time (DNT). The second outcome was the number of thrombolyses.

Stroke severity on admission was prospectively assessed for each patient. During the 3 years of the study, the National Institutes of Health Stroke Scale (NIHSS) was the severity stroke scale. 8 Severity cutt-offs were mild (NIHSS: 0–5), moderate (NIHSS: 6–14), and severe (NIHSS: 15–42). 9 The primary stroke type was determined via imaging (ischemic vs. hemorrhagic). Reduced consciousness at admission was defined by a Glasgow Coma Scale score of ≤ 10. Length of stay was recorded.

Patient demographics and medication use were registered at admission. The stroke risk factors registered were previous cerebrovascular disease (transient ischemic attack or stroke), myocardial infarction, treated hypertension or diabetes, dyslipidemia, overweight/obesity, any cancer diagnosis, alcohol use, and current smoking status. Atrial fibrillation was considered present if previously diagnosed or shown on electrocardiogram during admission or stay. Level of function poststroke at discharge was graded using the modified Rankin Scale (mRS), and patients were classified as being independent by an mRS score ≤ 2. 9 10

Complications related to intravenous thrombolysis have been reported. Symptomatic hemorrhagic transformation was defined when it was associated with a worsening of four or more points in NIHSS. Other complications investigated were orolingual angioedema and symptomatic hypotension.

### Statistical analysis

A descriptive analysis was made. The Shapiro-Wilk test was used to determine the normality of quantitative variables, which were then described using the median and percentiles. For the analyses of the door-to-CT time and DNT, we choose to add the mean because it is classically used. Categorical variables were presented as frequency and percentage. The IBM SPSS Statistics for Windows, version 21.0 (IBM Corp., Armonk, NY, USA) was used for all analyses.

### Ethical considerations

Consent was obtained prior to recruitment with permission for data use.

## RESULTS

**Figure 1:**
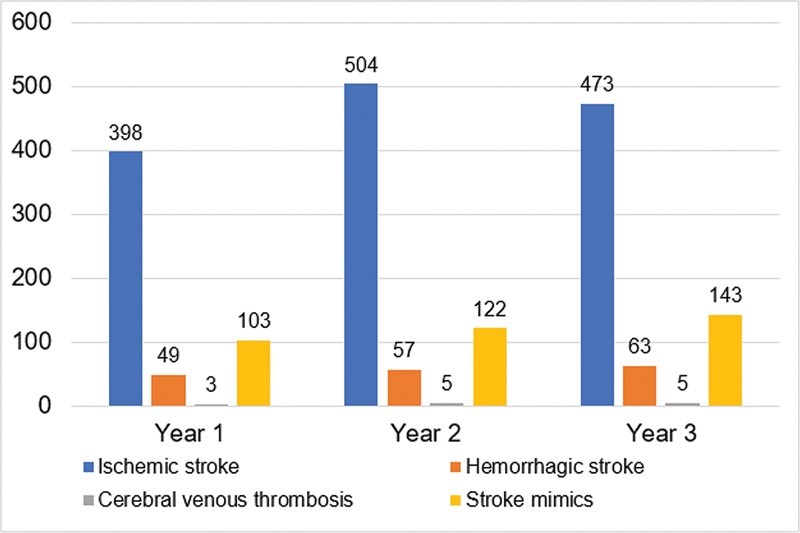
Distribution of most common diagnosis in stroke unit at Brazil's Central Arid Northeast on the first 3 years, from July 2018 to July 2021.

A total of 1,925 patients were included in the analysis: 553 patients in year 1, 688 in year 2, and 684 in year 3. The distribution of the most common diagnoses in the SUC along those 3 years are displayed in Figure 1 .

There were 1,374 ischemic-stroke patients. Most were men, with a mean age of 69.9. The most common risk factors were hypertension, being a current smoker, and presence of diabetes. Demographics, risk factors, and clinical features of these patients are exposed in Table 1 .

**Table 1:** Epidemiological profile of 1,374 stroke patients from the stroke unit in Brazil's Central Arid Northeast from July 2018 to July 2021

Characteristics	N (%)	Median
Total	1,374 (100)	–
Age in years, mean (SD)	–	71 (61-80) ^#^
Male sex	782 (56.9)	–
Hypertension	985 (71.68)	–
Current smoker	422 (30.71)	–
Diabetes	384 (27.9)	–
Prior stroke	292 (21.25)	–
Antiplatelets/anticoagulants	257 (18.7)	–
Cardiopathy	248 (18)	–
Alcohol user	235 (17.1)	–
Dyslipidemia	224 (16.3)	–
Overweight/obesity	217 (15.8)	–
Cancer	9 (0.7)	–

Notes: ^#^ (25 ^th^ –75 ^th^ percentile).

In the 1 ^st^ year, 130 patients arrived in the therapeutic time window. In the next year, it was 219 patients and, in the 3 ^rd^ year, 270 patients. Mean and median time were described on Table 2 . There were 42 intravenous thrombolyses in the 1 ^st^ year, 100 in the 2 ^nd^ year and 114 in the 3 ^rd^ year. Of these, 28 (10.9%) intravenous thrombolyses were performed for patients with NIHSS between 0 and 5, but with deficits considered potentially disabling, and 228 (89.1%) intravenous thrombolyses were performed for patients with severity stroke moderate or severe. The main reasons for not performing intravenous thrombolysis were stroke mimics, minor non-disabling deficits, large ischemia, or hemorrhage on admission CT scan. The comparison of service performance over the 3 years is described in Table 2 .

**Table 2:** Comparison of performance in stroke unit at Brazil's Central Arid Northeast for the first 3 years, from July 2018 to July 2021

	Year 1	Year 2	Year 3
Patients in therapeutic time window (n/%) ^*1^	130/23.5	219/31.8	270/39.5
Door-to-computed tomography time (minutes)			
Mean	14	11	13.3
Median	14 (10–20) ^#^	11 (8–15) ^#^	9.6 (7–12) ^#^
Door-to-needle time (minutes)			
Mean	44.6	26.8	20.3
Median	39.5 (29.5–60.8) ^#^	22 (17–30) ^#^	17 (14–22) ^#^
Thrombolysis (n/%) ^*2^	42/10.5	100/19.8	114/24.1

Notes ^*1^ :Considering all patients admited ^*2^ ;Considering only patients with ischemic stroke; ^#^ (25 ^th^ –75 ^th^ percentile).

Six patients (2.3%) had major complications due to thrombolysis: 1 orolingual angioedema, without clinical repercussion, and 5 symptomatic hemorrhagic transformations.

**Figure 2:**
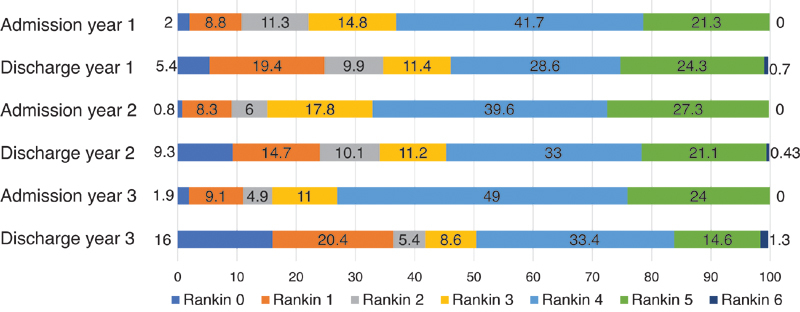
Percentage of Modified Rankin Scale distribution at Admission and Discharge in stroke unit at Brazil's Central Arid Northeast on the first 3 years, from July 2018 to July 2021.

**Figure 3:**
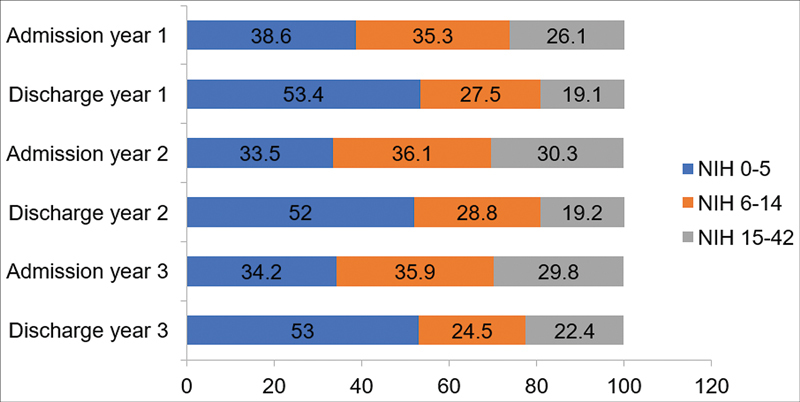
Percentage of NIH score distribution at Admission and Discharge in stroke unit at Brazil's Central Arid Northeast on the first 3 years, from july 2018 to july 2021.

The median hospitalization time was 10.5 days, 9.4 days, and 8.3 days in year 1, 2, and 3, respectively. The Rankin and NIHSS scales of admission and discharge during those 3 years are presented in Figures 2 and 3 . Currently, the in-hospital mortality rate of ischemic stroke in our SUC is 9.05%.

## DISCUSSION

Age, gender, and the most frequent risk factors were aligned with previous reports on demographic findings. 11

According to worldwide epidemiology, our DNT is better than average. 12 13 14 Our data shows lower mortality with better times than average due to considerable work done to improve DNT in our unit. 15

The most effective strategies include prenotification of arrival by emergency medical services (EMS), single-call activation of the stroke team, postponement of the registration process, going straight to CT on EMS stretcher, and administration of alteplase in the scanner as reported by other places. 12 We also accomplished neurologist acquisition on duty every day, acquisition of neurosurgeon staff and team training, since the second year.

Our in-hospital mortality rate was lower than previous reports in Brazil, such as the Botucatu stroke unit, with 12.7% on discharge, and others, with measure at 6 to 12 months from stroke (25%), but higher than Germany, with 5.4%. 16 17

There was an increase of 23.8% in SUC demand, with higher proportion of thrombolyses in the 2 ^nd^ year, probably due to prehospital education. Training was carried out for hospital and emergency room teams from most of the countryside cities for which we are the reference. During stroke protocol, reception, transportation assistant, radiology and stroke unit teams, all gather forces and stay alert for the arrival of the patient. In spite of the pandemic in the third year, our proportion of thrombolyses still improved.

The present study had some limitations, such as the lack of information of asymptomatic hemorrhagic transformations after thrombolysis, premorbid Rankin scale, mortality rate 90 days after discharge, and some lost data among cases. However, this was the first report of stroke data in Brazil's Central Arid Northeast.

In conclusion, it is a fact that the SUC improves the quality of care to users due to significant reduction of the sequelae generated by the disease and its mortality rate.

Hospital participation in a multidimensional quality initiative was associated with improvement on alteplase administration time.

There are many exciting areas of future direction, including reduction of DNT by improvement of prehospital response times and acquisition of endovascular treatment, to accomplish an even better outcome.
